# Statin intensity, prescribing patterns, and LDL‑C reduction following ACS and in CCD patients who underwent PCI in a tertiary care setting

**DOI:** 10.1186/s12872-026-05874-x

**Published:** 2026-05-05

**Authors:** Sara Abdelrady, Seeba Zachariah, Sony Mathew, Karim Ghannem

**Affiliations:** 1 Department of Pharmacy Practice, Gulf Medical University, Ajman, UAE; 2 Department of Cardiology, Thumaby University Hospital, Ajman, UAE; 3 College of Medicine, Gulf Medical University, Ajman, UAE

**Keywords:** Acute coronary syndrome, Atherosclerotic cardiovascular disease, Lipid-lowering therapy, Statin, Secondary prevention

## Abstract

**Background:**

Atherosclerotic cardiovascular disease (ASCVD) remains a major cause of morbidity and mortality, with dyslipidemia representing a key modifiable risk factor in secondary prevention. Despite clear guideline recommendations, real‑world lipid‑lowering therapy (LLT) use and LDL‑C target attainment remain suboptimal in many regions. This study evaluated LLT prescribing patterns and LDL‑C reduction among patients admitted with acute coronary syndrome (ACS) or undergoing percutaneous coronary intervention (PCI) for chronic coronary disease (CCD) in a tertiary care center.

**Methods:**

This retrospective observational study included adult patients hospitalized between June 2020 and June 2024 for ACS or elective PCI for CCD. Electronic medical records were screened by independent chart review. Demographics, cardiovascular risk factors, LLT prescriptions, and lipid profiles at baseline and 6 (± 3) months were collected. LLT regimens and the reduction in LDL were analyzed. Non-normally distributed continuous variables were analyzed using non‑parametric methods Wilcoxon Signed Ranks Test and Kruskal–Wallis test, while categorical variables were evaluated using Chi-Square test.

**Results:**

Of 363 screened patients, 314 met inclusion criteria, and 98 had follow‑up lipid measurements at 6 (± 3) months. Statin monotherapy was prescribed in 81.5% of patients at discharge, while 12.4% received combination therapy. High‑intensity statins were predominantly used, although some patients received only moderate‑intensity therapy. Prior statin exposure significantly influenced discharge prescribing patterns (*p* < 0.001). Significant LDL‑C reductions were observed at 6 (± 3) months among patients with and without prior LLT (*p* < 0.001). Overall, 47.95% achieved LDL‑C target of < 70 mg/dL, others needed improvements in their adherence and therapy optimization.

**Conclusion:**

In the patient cohort studied, prescribing pattern were largely consistent with guideline recommended statin therapy. Although all treatment groups achieved significant reductions in the LDL‑C levels, a proportion of patients remained above guideline‑recommended targets, reflecting need for exploring additional strategies to optimize lipid control. Continued follow‑up and broader implementation of guideline‑directed LLT intensification are warranted to improve LDL‑C control in post‑ACS and post‑PCI CCD populations.

**Trial registration:**

Not applicable.

## Introduction

Atherosclerotic cardiovascular diseases (ASCVD) remain a major global cause of mortality and morbidity [1]. The burden of ASCVD continues to rise, with an estimated 315 million cases of coronary artery disease (CAD) reported in 2022 [2]. The Middle East has among the highest rates of cardiovascular–related mortality [3], and in the United Arab Emirates (UAE), many post-acute coronory syndrome (ACS) patients present with multiple cardiometabolic risk factors, with dyslipidaemia consistently classified as ahigh-risk ASCVD feature [3–5]. The prevalence of established ASCVD among patients with type 2 diabetes approaches one-third in the UAE and one-fifth in Middle Africa [6]. Modifiable risk factors - particularly dyslipidaemia - play a central role in secondary prevention after ACS or chronic coronary disease (CCD) patients underwent PCI, underscoring the need for intensive lipid optimisation to improve continuity and quality of care [7].

Hyperlipidaemia remains common among ACS patients, including those already receiving statins. In a prospective study of Egyptian ACS patients, statin therapy was intensified post-event, yet LDL-C levels remained persistently elevated, highlighting challenges in achieving lipid targets [8]. Reducing LDL-C lowers ASCVD risk [9], and multiple studies support risk stratification and LDL-C goal optimisation. However, only 27.7% of Emirati patients achieved LDL-C targets 6 months after ACS [10]. ESC guidelines recommend initiating high-intensity statins in all ACS patients, targeting LDL-C < 55 mg/dL and ≥ 50% reduction from baseline [9], while American guidelines recommend < 70 mg/dL [11]. When goals are unmet, ezetimibe and subsequently PCSK9 inhibitors should be added [11, 12]. For patients with recurrent ASCVD events, an LDL-C goal of 40 mg/dL is advised [13]. Non-statin therapies are also recommended for statin-intolerant ACS patients to reduce major adverse cardiovascular events (MACE) [11–14].

The UAE ACS patients tend to be younger, with diabetes and smoking as common risk factors [4]. A retrospective study in Dubai showed that 60.40% of patients had LDL-C ≥ 70 mg/dL despite 93.45% receiving LLT; high-intensity statins were used in 67.79%, statin plus ezetimibe in 4.55%, and PCSK9 inhibitors in 0.20% [5]. Suboptimal lipid control was also reported in post-CABG patients, where 93.1% received high-intensity statins at discharge, yet only 59.3% achieved LDL-C < 70 mg/dL and 29% achieved < 55 mg/dL [3, 15].

Guideline-directed LLT remains essential to reduce ASCVD burden and ensure adequate LDL-C control [7]. Patients with chronic coronary disease (CCD) who undergo PCI fall within the same very-high-risk ASCVD category and require similarly aggressive LDL-C management [7, 16]. Including CCD post-PCI patients is therefore clinically relevant, as they require equally aggressive lipid-lowering strategies to prevent recurrent events. In post-STEMI patients, rosuvastatin 20 mg (70%) and atorvastatin 40 mg (12.5%) were commonly used, yet fewer than half achieved lipid targets [17]. Similar suboptimal LLT use was reported in Japan, underscoring the need for therapy intensification [18]. The objective of this study was to evaluate lipid‑lowering therapy use among post‑ACS and CCD patients who underwent PCI patients in a clinical practice and assess LDL‑C reduction after 6 months.

## Methods

### Study design and population

This retrospective observational study included adult patients treated at our institution between June 2020 and June 2024. The overall study duration was 1 year, from June 2024 to June 2025. Electronic medical records were queried using ICD‑10 codes I20, I21, I23, I24, I25, and Z95.5 to identify individuals with angina, acute myocardial infarction, acute ischemic heart disease, chronic ischemic heart disease, and prior coronary angioplasty with stent placement. These codes served as an initial filter to capture all potentially eligible cases.

An independent chart review was conducted to confirm clinical diagnoses and ensure accurate classification. Patients were included if they met criteria for either: Admission for acute coronary syndrome (ACS)—including ST‑elevation myocardial infarction (STEMI), non‑ST‑elevation myocardial infarction (NSTEMI), or unstable angina (UA); or Admission for elective percutaneous coronary intervention (PCI) performed for chronic coronary disease (CCD).

CCD patients undergoing PCI were intentionally included because both ACC/AHA and ESC guidelines classify them as very‑high‑risk ASCVD, with lipid‑lowering therapy recommendations equivalent to those for post‑ACS patients. Their comparable long‑term risk of recurrent cardiovascular events justifies evaluating both groups within a unified high‑risk cohort.

### Data collection

For all eligible patients, demographic characteristics, cardiovascular risk factors, lipid‑lowering therapy (LLT) prescriptions, and lipid profiles at baseline and follow‑up were extracted. LLT regimens were categorized as: high‑intensity statin monotherapy, statin–ezetimibe combination therapy, or statin plus PCSK9 inhibitor therapy.

Follow‑up LDL‑C measurements were collected at approximately 6 (± 5) months after the index ACS event or PCI. This window reflects routine clinical practice, where lipid testing is typically performed within the first several months after initiating or adjusting LLT. The ± 5‑month allowance accounts for natural variability in outpatient scheduling while ensuring that lipid values represent medium‑term treatment response rather than acute peri‑hospitalization fluctuations.

### Exclusion criteria

Patients were excluded if they: had missing or incomplete clinical or lipid data, were long‑term care or chronically institutionalized patients, died during the index hospitalization, were immediately transferred to another healthcare facility, or left against medical advice, preventing adequate follow‑up or assessment of LLT use.

### Ethical considerations

The study was conducted in accordance with the principles of the Declaration of Helsinki. Ethical approval was obtained from the Gulf Medical University Institutional Review Board prior to data collection. As this was a retrospective review of existing medical records, the requirement for informed consent was waived by the Institutional Review Board. All patient data were anonymized and handled confidentially to ensure privacy and compliance with institutional and national regulations.

### Statistical analysis

Baseline characteristics were summarized using descriptive statistics. Categorical variables were analyzed using Chi-Square test. Continuous variables, including age and LDL‑C levels, were assessed for normality using the Shapiro–Wilk test, which demonstrated non‑normal distributions. Consequently, continuous variables are presented as medians with interquartile ranges (IQR), and non‑parametric methods were applied for all related analyses. Changes in LDL‑C values over time within the same patient group were evaluated using the Wilcoxon signed‑rank test and Kruskal–Wallis test. All statistical tests were two‑sided, and a p‑value < 0.05 was considered statistically significant. Data analysis was conducted using IBM SPSS Statistics, version 30.

## Results

A total of 363 patients were screened during the study period, of whom 314 met the inclusion criteria and were included in the final analysis. Forty‑nine patients were excluded: 33 for not meeting inclusion criteria, 16 due to missing clinical data resulting from immediate transfer to another healthcare facility or leaving against medical advice, 4 were long‑term care patients, and 3 died during hospitalization. Of the 314 patients included at baseline, 98 had available follow‑up lipid measurements at 6 (± 3) months. Median age was 48 years with 92.4% males. Baseline charecteristics of the study population is shown in Table 1.

**Table 1:** Baseline charecteristics of the Patients

Variable	Category	Number of patients (%) (*N* = 314)
Gender	Male	290 (92.4%)
	Female	24 (7.6%)
BMI	≥ 30 kg/m^2^	221 (70.4%)
	< 30 kg/m^2^	93 (29.6%)
Ethnicity	Middle Eastern	66 (21.0%)
	South Asian	229 (72.9%)
	Other Asian	9 (2.9%)
	African	10 (3.2%)
Insurance status	No	53 (16.9%)
	Yes	261 (83.1%)
Alcohol use	No	313 (99.7%)
	Yes	1 (0.3%)
Smoking	Non-Smoker	243 (77.4%)
	Ex-smoker	6 (1.9%)
	Current smoker	65 (20.7%)
Prior Lipid Lowering Therapy	No	194 (61.8%)
	Yes	120 (38.2%)
Cardiovascular Comorbidities	Dyslipidemia	109 (34.7%)
	Hypertension	143 (45.5%)
	Diabetes	86 (27.4%)
	Chronic Kidney Disease	5 (1.6%)
	Previous ACS	23 (7.3%)
	Previous PCI	7 (2.2%)
	Ischemic Heart Disease	55 (17.5%)
	Stroke	3 (1.0%)
	Heart failure	5 (1.6%)
	None of the above comorbidities reported	76 (24.2)
Diagnosis for hospital admission	ACS	266 (84.7%)
	Elective PCI	48 (15.3%)

**Fig. 1:**
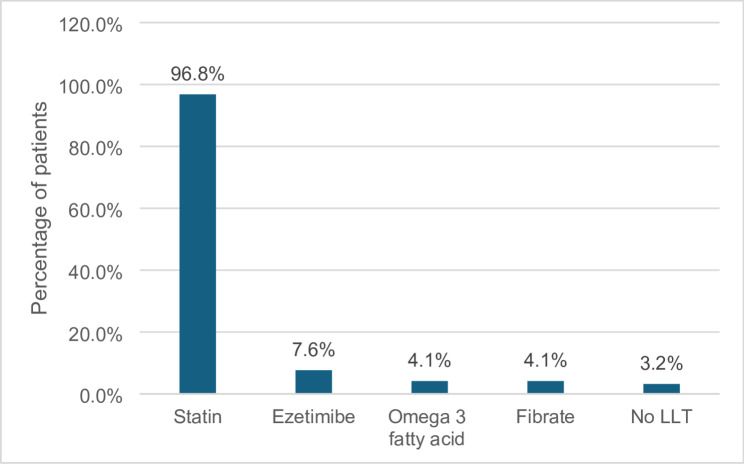
Lipid lowering therapy use among the patient population (*N* = 314)

The percentage of LLT use at hospital discharge is shown in Fig. 1. Statin use was predominant among the patients studied. Among those who received lipid‑lowering therapy at discharge, most were prescribed statin monotherapy (81.5%), while 12.4% received combination therapy, as shown in Table 2.

**Table 2:** Distribution of monotherapies and combination therapies (*N* = 314)

LLT	Number	%
Any Statin therapy (mono or combination)	304	93.5%
Statin Monotherapy	265	81.5%
Combination therapy	39	12.4%
Statin + Ezetimibe﻿	17	5.4%
Statin + Omega 3 FA	7	2.2%
Statin + Fibrate	6﻿	1.9%
Statin + Fibrate + Omega 3 FA	3	1.0%
Statin + Ezetimibe + Omega 3 FA	2	0.6%
Statin + Ezetimibe + Fibrate	3	1.0%
Statin + Ezetimibe + Omega 3 FA + Fibrate	1	0.3%﻿
No lipid lowering therapy	10	3.2%

*FA* Fatty Acid

**Fig. 2:**
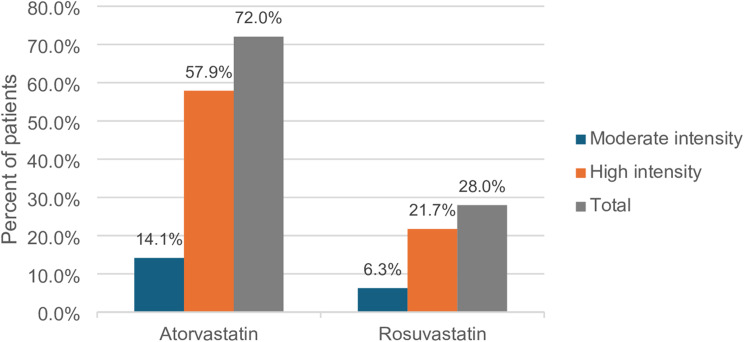
Distribution of use of atorvastatin and rosuvastatin (*n* = 304)

Use of moderate‑ and high‑intensity atorvastatin and rosuvastatin is illustrated in Fig. 2. Although high‑intensity statins were used in most patients, some still received only moderate‑intensity statins.

The Kruskal–Wallis test showed that LDL-C level was higher for patients with prior exposure to LLT having median and interquartile range 122.0 (99.0-147.0) compared to those who did not have LLT before, 101.3 (79.0-130.0). This difference was statistically significant (*p*-value 0.001). Regardless, a significant difference (*p*‑value < 0.001) was observed in LDL‑C reduction among patients who received LLT at the study site over 6 (± 3) months. Both patients without prior LLT and those with prior LLT at baseline demonstrated significant reductions in LDL‑C levels at 6 (± 3) months. Prior statin exposure played a critical role and was the strongest predictor of the discharge regimen. Clinicians showed a clear tendency to continue the pre‑admission statin type and intensity. Among patients previously treated with atorvastatin, 31.1% continued the same agent, whereas 8.2% were switched to rosuvastatin. Prior statin treatment was significantly associated with the prescription of atorvastatin and rosuvastatin at discharge (*p* < 0.001). Patients receiving atorvastatin were more likely to have been previously treated with atorvastatin (31.1% vs. 8.2%, p-value ), while prior rosuvastatin use was more common among those currently prescribed rosuvastatin (29.4% vs. 6.4%, p-value ). While both moderate‑intensity and high‑intensity statins significantly reduced LDL‑C levels, high‑intensity statins were preferred. Additional details, including LDL‑C reductions in both acute and chronic coronary conditions, are presented in Table 3.

With LLT at the study site 47 out of 98 (47.95%) reached their target LDL-C below 70 mg/dL, while 31.6% reached their LDL-C below 55 mg/dL. Very high risk population was not identified in this study.

**Table 3:** Six months LDL-C reduction charecterestics among study population

Variable	LDL-C at baseline Median (IQR)	LDL-C at 6(± 3) months Median (IQR)	x Z -value	*p*- value*	Effect size (*r*)	*N* (paired)
Total patient received LLT at Site (*n* = 314)	116.0 (86.0-144.0)	74.0 (49.4-102.2)	-6.25	< 0.001	0.65	94
Patients without prior LLT (*n* = 194)	122.0 (99.0-147.0)	66.0 (47.0- 100.0)	-5.23	< 0.001	0.71	54
Patients with prior LLT (*n* = 120)	101.3 (78.9-131.8)	77.7 (51.5- 114.5)	-3.58	< 0.001	0.57	40
Moderate intensity statin (*n* = 61)	102.3 (78.4-129.3)	75.5 (44.9-112.1)	-2.33	0.02	0.53	19
High intensity statin (*n* = 243)	121.0 (89.9–147.0)	70.8 (50.8-100.4)	-5.74	< 0.001	0.67	73
Atorvastatin (*n* = 219)	117.8 (89.6–145.0)	74.0 (53.0-100.0)	-5.12	< 0.001	0.62	68
Rosuvastatin (*n* = 85)	111.0 (80.6-143.8)	74.0 (40.9-112.5)	-3.43	< 0.001	0.70	24
ACS (*n* = 266)	117.5(90.0-144.0)	71.0(47.5–100.0)	-5.87	< 0.001	0.68	75
CCD with PCI (*n* = 48)	95.00(79.3-143.5)	80.00(53.0-115.0)	-2.13	0.03	0.49	19

*LDL-C* Low density lipoprotein - Cholesterol, *LLT*  Lipid Lowering Therapy, *ACS*  Acute Coronory Syndrome, *CCD*  Chronic Coronory Disease, *PCI*  Percutaneoud Coronory Intervention

*Wilcoxon Signed Ranks Test

## Discussion

Statin therapy remains a cornerstone of secondary prevention in coronary artery disease, effectively reducing atherosclerotic plaque burden, lowering cardiovascular event risk, and decreasing the need for revascularisation. In our cohort of patients with ACS, the median age was 48 years, with males predominating the population, reflecting a younger, predominantly male profile compared with other regional studies [10]. A retrospective study of post‑ACS patients found that 58% of Emirati patients were on prior LLT at admission, indicating high cardiovascular risk [10]. In comparison, approximately 32.3% of patients in our cohort were on LLT upon admission.

Most patients in this study received statin monotherapy, consistent with previous findings [3]. Atorvastatin was the most commonly prescribed statin, followed by rosuvastatin in both studies. Prior research has highlighted a statin‑focused approach, with recommendations to transition toward more contemporary guideline‑directed therapy [3, 10]. As a result of current prescribing patterns, LLT was not optimally intensified, and many patients remained above LDL‑C targets due to underutilisation of combination therapy and limited escalation. Enhanced follow‑up, monitoring, and LLT intensification strategies are needed to fully implement guideline recommendations, particularly in high‑risk patients with elevated baseline LDL‑C. These findings highlight opportunities for LLT optimisation and areas for future investigation.

International guidelines emphasise early and intensive therapy. Early initiation of PCSK9 inhibitors has demonstrated significant reductions in atherosclerotic burden, with patients achieving LDL‑C targets within a median of 37 days after hospitalisation [19]. As per the 2026 AHA dyslipidemia guideline, in secondary prevention, a goal of LDL-C < 55 mg/dL and non–HDL-C < 85 mg/dL is recommended for those at very high risk of ASCVD events. The majority of patients with a history of ASCVD events likely qualify for an LDL-C goal of < 55 mg/dL. Only a smaller number of patients with ASCVD not at very high risk have an LDL-C goal of at least < 70 mg/dL [20]. Only 30% of ACS patients in Europe achieved LDL‑C < 70 mg/dL within one year [21]. Local protocols are needed to improve in‑hospital and post‑discharge LLT adherence [21]. Combination therapy with ezetimibe has shown superior LDL‑C reduction compared with statin monotherapy [22].

In Saudi Arabia, only 15.9% of patients achieved LDL‑C < 55 mg/dL per ESC targets [23]. A multicentre study evaluating early triple therapy (high‑intensity statin, ezetimibe, PCSK9 inhibitor) reported 92% achieving LDL‑C < 55 mg/dL at 6 months, demonstrating feasibility and effectiveness in very high‑risk ACS patients [24]. Italian data similarly showed improved LDL‑C attainment after targeted educational interventions, with 35% achieving < 55 mg/dL and 50% reaching target within 120 days [25]. Overall, observational studies consistently show suboptimal LLT use and poor LDL‑C goal attainment [26, 27]. The Gulf RACE‑3Ps registry reported a mean age of 52.7 years and 90% male representation [28]. Only 12.1% of patients in a previous study achieved their target LDL-C levels [29], whereas 47.9% of patients in our cohort achieved the ACC/AHA‑recommended LDL-C target of < 70 mg/dL. Another UAE study reported that 60.4% of patients presented with LDL‑C ≥ 70 mg/dL, indicating suboptimal lipid control despite concomitant LLT, particularly in high‑risk patients [7]. In our cohort, 52% of patients remained above target despite guideline recommendations emphasising LDL-C target achievement and LLT optimisation. Further research is needed to identify barriers to achieving guideline‑recommended lipid targets.

Median LDL-C reductions were observed with rosuvastatin (111 to 74 mg/dL) and atorvastatin (117.5 to 75 mg/dL). All groups demonstrated significant LDL-C reductions toward guideline‑recommended targets, supporting the need for further intensification with add‑on therapy such as ezetimibe or fibrates. A previous UAE study reported that 27.7% achieved LDL‑C < 70 mg/dL, while another study from Dubai showed that approximately 40% achieved target LDL‑C in the first year post‑MI. Our study demonstrated the highest LDL‑C attainment, with approximately 48% achieving target LDL‑C 6 months post‑ACS/PCI. This may reflect the higher proportion of patients discharged on high‑intensity statins and the absence of low‑intensity statin use, consistent with guideline recommendations and clinical preference.

Despite these improvements, LDL‑C goals remain unmet for many high‑risk patients. Future studies should investigate potential causes, including underutilisation of combination therapy and limited escalation in clinical practice. Greater use of non‑statin therapies, such as ezetimibe, bempedoic acid, icosapent ethyl, and PCSK9 inhibitors should be encouraged in accordance with guidelines. Further research is needed to identify factors contributing to suboptimal LDL‑C control, including patient adherence and the impact of structured counselling. Implementing hospital‑based assessment tools to monitor guideline adherence and escalate therapy according to LDL-C targets may help optimise LLT in post‑ACS patients.

### Limitations

This study has several limitations. Due to its retrospective design, key variables such as patient adherence to medication, lifestyle modifications, and control of other cardiovascular risk factors were not captured, all of which may influence LDL‑C levels and target attainment. A substantial proportion of patients did not return for follow‑up lipid monitoring, consistent with trends reported in other studies from the Middle East region. Additionally, the analysis did not explore underlying reasons for discharge without lipid‑lowering therapy, including potential contraindications, adverse effects, or statin intolerance. Subgroup analysis of LDL-C reduction below 55 mg/dL for the very high-risk poplation was not performed. The study cohort may not be representative of the broader UAE population, limiting generalisability. Future research should incorporate prospective follow‑up and assess adherence‑related factors to better understand long‑term lipid management outcomes.

## Conclusion

Among 314 patients studied, statin monotherapy remained the predominant LLT at discharge, with high‑intensity statins used in most patients, although a subset who were already on moderate intensity statin continued to receive the same therapy. Prior statin exposure strongly influenced discharge prescribing patterns, with clinicians largely continuing the same agent and intensity. Despite these patterns, LDL‑C levels showed significant reductions over 6 (± 3) months among patients receiving LLT, including those with and without prior therapy. Approximately 48% achieved LDL-C < 70 mg/dL and only 31.6% reached < 55 mg/dL, more collaborative efforts are required. These findings highlight the need for more consistent guideline‑directed LLT intensification, improved follow‑up, and broader adoption of non‑statin therapies to optimise LDL‑C reduction in post‑ACS and post‑PCI CCD populations.

Future studies should adopt multicentre prospective designs and incorporate patient adherence to LLT as a key variable. Also, LDL-C reduction below 55 mg/dL for very high-risk population is to be studied further. Beyond evaluating statin intensity and baseline cardiovascular risk, there is a clear need to expand the use of non‑statin therapies with proven cardiovascular benefit, particularly in patients who do not achieve target LDL-C levels or who have statin intolerance.

## Data Availability

Data are included in the article; additional data are available on request to the corresponding author.

## Abbreviations

ACC/AHA: American College of Cardiology/American Heart Association
ACS: Acute Coronary Syndrome
ASCVD: Atherosclerotic Cardiovascular Disease
BMI: Body Mass Index
CABG: Coronary Artery Bypass Grafting
CAD: Coronary Artery Disease
eGFR: Estimated Glomerular Filtration Rate
EMR: Electronic Medical Record
ESC: European Society of Cardiology
HDL‑C: High‑Density Lipoprotein Cholesterol
IQR: Interquartile Range
LDL‑C: Low‑Density Lipoprotein Cholesterol
LLT: Lipid‑Lowering Therapy
MACE: Major Adverse Cardiovascular Events
NSTE‑ACS: Non‑ST‑Elevation Acute Coronary Syndrome
PCI: Percutaneous Coronary Intervention
PCSK9i: Proprotein Convertase Subtilisin/Kexin Type 9 Inhibitor
STEMI: ST‑Elevation Myocardial Infarction
TG: Triglycerides
